# Results of the National External Quality Assessment for Toxoplasmosis Serological Testing in China

**DOI:** 10.1371/journal.pone.0130003

**Published:** 2015-06-12

**Authors:** Kuo Zhang, Lunan Wang, Guigao Lin, Yu Sun, Rui Zhang, Jiehong Xie, Jinming Li

**Affiliations:** 1 National Center for Clinical Laboratories, Beijing Hospital, Beijing, China; 2 Graduate School, Peking Union Medical College, Chinese Academy of Medical Science, Beijing, China; NIH, UNITED STATES

## Abstract

**Background:**

Toxoplasmosis is typically diagnosed by serologic testing. External quality assessment (EQA) of clinical laboratories could ensure the accuracy and reliability of serological tests. We assessed the quality of toxoplasma serological assays in Chinese clinical laboratories by an EQA performed between 2004 and 2013 by the National Center for Clinical Laboratories.

**Methodology and Findings:**

EQA panels were prepared and shipped at room temperature to participating laboratories that employed toxoplasma IgG and IgM serological detection. By 2013, 5,384 EQA test reports for toxoplasma-specific IgM and 2,666 reports for toxoplasma-specific IgG were collected. Enzyme-linked immunosorbent (ELISA) and chemical immunofluorescent assays were the most commonly used detection methods. The overall coincidence rates of negative samples were better than those of positive samples. The overall EQA score for toxoplasma-specific IgM detection ranged between 84.3% and 99.6%. The ratio of laboratories that achieved correct IgG detection ranged from 61.1% to 99.3%. However, the inter- and intra-assay variabilities were found to be considerable. The most common problem was failure to detect low titers of antibody.

**Conclusion:**

The EQA scheme showed an improvement in toxoplasma serological testing in China. However, further optimization of assay sensitivity to detect challenging samples remains a future challenge.

## Introduction

*Toxoplasma gondii* is a protozoan obligate intracellular parasite that causes the disease toxoplasmosis. It has been estimated that 10% to 70% of the world’s population is infected by *T*. *gondii*, and the general infection rate of *T*. *gondii* in the northeast and south of China was found to be 12.3% [1]. Although the infection is generally asymptomatic or results in a clinical disease that is not recognized, it can cause severe health problems in individuals who are immunocompromised such as congenitally infected infants, transplant recipients, and AIDS patients. Infection of pregnant woman can lead to abortion, hydrocephalus, cerebral calcification, and/or chorioretinitis [2]. Therefore, programs aimed at detecting *T*. *gondii* infection in pregnant women by systematic screening have been performed in several countries such as France and Austria [3]. Serologic diagnosis, based on the detection of toxoplasma-specific immunoglobulin (Ig) G and IgM antibodies in the serum, is often used to determine the immune status of patients, and prenatal screening for antibodies is routine practice in many parts of the world [4]. As non-specific clinical manifestations can complicate diagnosis of the disease and because of the importance of early detection of *in utero* infection, accurate diagnostic tests are essential. Therefore, a number of more sensitive methods, such as a serum IgG avidity test, PCR, and western blotting using serum from mother-baby pairs, have been developed [5]. However, routine screening of toxoplasma-specific IgG and IgM in serum is still mostly used in clinical laboratories, especially in China [1, 5].

Quality assurance of serologic testing is important to ensure accurate and reliable screening of susceptible individuals, especially for pregnant women. Therefore, laboratories should participate in external quality assessment (EQA) schemes conducted by independent organizations [6]. Participation in relevant EQA schemes allows for comparing test results between different clinical laboratories, it provides insight into national performance levels, and it allows for improving national performance levels [7]. An EQA program for the evaluation of clinical toxoplasma IgG and IgM serological detection assays in China, was established in 2004 by the National Center for Clinical Laboratories (NCCL). The program allowed clinical laboratories to review their testing process. Throughout the EQA, potential issues associated with the serological tests were identified. The aim of the present study was to assess the quality of toxoplasma serological tests in Chinese clinical laboratories. We based our study on data gathered between 2004 and 2013.

## Materials and Methods

### Panel preparation and distribution

The toxoplasma EQA scheme consisted of two distributions of human serum specimens for the examination of toxoplasma IgG and IgM antibodies with a request to report qualitative results twice a year (in May and October). Each distribution consisted of two panels of serum, one to test for toxoplasma IgG, another to test for toxoplasma IgM. Each panel consisted of five serum specimens. The panels were majorly prepared using sera pools of blood donors kindly donated by Shenzhen blood center and Liaoning blood center in China or minor sera pools donated by the company Viron/Serion (Würzburg, Germany) or minorly purchased from Guangzhou Kang Run biological products development co., LTD (Guangzhou, China) (Lot:L3720,W84,W64,W37). The NCCL tested the specimens for toxoplasma-specific IgG and IgM antibodies prior to dispatch, using several commercial enzyme-linked immunosorbent assays (ELISA) or chemiluminescence immunoassay (CIA) kit for toxoplasma IgG and IgM. The tests used to produce the EQA panel of sera for each year and precisely detail for positive sera were shown in Tables 1 and 2. The samples with lower titer (low S/CO ratio) detected by at least three of these kits were classified as weakly positive samples. The titers of negative sera provided were all smaller than cutoff of kits(data not shown). The specimens were divided in screw cap centrifuge tubes and stored at -40°C until distribution. The EQA panels, containing toxoplasma IgG- and IgM-positive specimens as well as negative specimens, were shipped at room temperature (18–25°C) by express mail service (shipment time is about 1–3 days)to participating laboratories, together with EQA scheme instructions for the proper handling of specimens and the reporting of results.

**Table 1:** Serological characterization of the samples by NCCL and coincidence rate by participants for the Detection of IgM.

		Virion-serion(EIA)		Trinity(EIA)		Medson(EIA)		Architect (CIA)	
Year	Samplecode	Titer by NCCL^a^	Positive coincidence rate^b^ (%)	Titer by NCCL^a^	Positive coincidence rate^b^ (%)	Titer by NCCL^a^	Positive coincidence rate^b^ (%)	Titer by NCCL^a^	Positive coincidence rate^b^ (%)
2013	1314	2.7	96.8(30/31)	1.5	93.5(29/31)	4.8	100(44/44)	5.8	100(11/11)
	1323	2.1	100(30/30)	1.5	100(32/32)	4.5	100(41/41)	5.3	100(10/10)
2012	1211	2.0	100(39/39)	1.8	100(29/29)	4.9	100(49/49)	6.3	100(8/8)
	1221	2.3	100(37/37)	2.7	92.9(26/28)	4.4	100(49/49)	7.8	100(4/4)
	1225	2.1	100(37/37)	1.8	82.1(23/28)	4.0	93.9(46/49)	5.8	100(4/4)
2011	1114	1.6	97.3(36/37)	1.3	100(16/16)	2.5	100(51/51)	4.7	100(3/3)
	1115	1.7	81.1(30/37)	1.0	93.4(15/16)	2.1	100(51/51)	3.7	100(3/3)
	1121	2.4	100(33/33)	1.9	100(15/15)	4.6	100(47/47)	5.8	100(2/2)
	1124	2.2	100(33/33)	1.8	86.7(13/15)	3.8	100(47/47)	4.7	100(2/2)
2010	1012	2.3	100(32/32)	1.7	100(16/16)	6.0	100(37/37)	—	—
	1013	1.2	93.8(30/32)	1.1	62.5(10/16)	3.0	100(37/37)	—	—
	1022	3.2	100(32/32)	2.9	100(17/17)	5.5	100(39/39)	—	—
	1023	5.3	100(32/32)	1.4	100(17/17)	4.5	100(39/39)	—	—
2009	0923	1.1	68.0(17/25)	1.2	60.0(6/10)	—	—	—	—

^a^ The cutoff was 1.00. The titers of all negative samples were smaller than 1.00 by different tests detected at NCCL (data not shown).

^b^ Positive coincidence rate of the participating labs using the same tests used to produce the EQA panel.

—,The sample was not detected by the test before dispatch and no participants used the test at that year.

**Table 2:** Serological characterization of the samples by NCCL and coincidence rate by participants for the Detection of IgG.

		Virion-serion(EIA)		Trinity(EIA)		Medson(EIA)		Architect (CIA)	
Year	Sample code	Titer by NCCL^a^	Positive coincidence rate^b^ (%)	Titer by NCCL^a^	Positive coincidence rate^b^ (%)	Titer by NCCL^a^	Positive coincidence rate^b^ (%)	Titer by NCCL^c^	Positive coincidence rate^b^ (%)
2013	1313	1.4	86.7(13/15)	1.8	100(29/29)	3.2	100(23/23)	4.7	100(5/5)
	1315	1.9	86.7(13/15)	2.0	100(29/29)	2.7	100(23/23)	8.5	100(5/5)
	1323	3.7	93.3(14/15)	3.4	100(29/29)	9.3	100(22/22)	9.4	100(7/7)
2012	1224	1.9	93.8(15/16)	2.6	92.0(23/25)	2.7	96.7(29/30)	3.9	100(2/2)
2011	1121	1.4	100(17/17)	1.1	43.5(10/23)	1.0	21.4(6/28)	3.1	100(2/2)
	1124	1.1	76.5(13/17)	1.1	34.8(8/23)	1.1	46.4(13/28)	3.0	100(2/2)
2010	1012	2.8	100(23/23)	2.0	95.0(19/20)	3.2	100(22/22)	—	—
	1013	4.0	100(23/23)	2.6	95.0(19/20)	5.2	100(22/22)	—	—
	1014	3.6	100(23/23)	2.6	95.0(19/20)	6.0	100(22/22)	—	—
	1025	4.1	100(21/21)	3.3	100(21/21)	7.9	100(23/23)	—	—

^a^ The cutoff was 1.00. The titers of all negative samples were smaller than 1.00 by different tests detected at NCCL (data not shown).

^b^ Positive coincidence rate of the participating labs using the same tests used to produce the EQA panel.

^c^ Concentration values ≥ 3.0 IU/mL areconsidered reactive for IgG antibodies to Toxoplasma gondii

—,The sample was not detected by the test before dispatch and no participants used the test at that year.

### Participants

Hospital laboratories from China that employ toxoplasma IgG and IgM serological detection were invited to participate in this EQA scheme. The participating clinical laboratories tested the EQA panels using their standard protocols and returned the test results to NCCL via a data submission form on the NCCL website. Participants using EIAs were asked to provide signal/cut-off (S/CO) values if possible.

### Evaluation of the results and statistical analysis

The EQA data were scored according to qualitative criteria; every correct positive or negative test result in accordance with the expected results of NCCL of the specimens in each panel was assigned a point of 20, while false negative or false positive results were not scored. The highest possible total EQA score per panel was thus 100 points, and an EQA points <80 was considered unqualified. Data were entered into Microsoft Excel spreadsheets and analyzed using Microsoft Excel and the Prism 6 software (GraphPad Software Inc., La Jolla, CA, USA) and SPSS 16.0(SataCorp).

## Results

### Participant laboratories

Chinese clinical hospital laboratories offering toxoplasma IgG/IgM testing were invited to participate in the EQA scheme. At the start of the IgM scheme in 2003, 79 laboratories participated and reported qualitative results. At the start of the IgG scheme in 2008, 94 laboratories were participating. The number of participating laboratories increased steadily over the past years. By September 2013, 843 laboratories had reported 2,666 toxoplasma-specific IgM test results, while 551 laboratories had submitted 5,384 EQA test reports for toxoplasma-specific IgG.

### Analysis of the toxoplasma-specific IgM scheme

All IgM test results, acquired by the different routine detection assays as used by the participating hospital laboratories, were analyzed and scored (Table 3). The ratio of laboratories that correctly identified all five (EQA score = 100) samples within a panel ranged from 45.3% (panel 071) to 95.9% (panel 082). The overall coincidence rates for negative samples (98.4%) were higher than for positive samples (86.7%). A very low ratio of laboratories with an EQA score of 100 (45.3%) was observed for panel 071. Seventy-three laboratories (42.5%) failed to identify two positive samples within this panel, and 21 participants (12.2%) misidentified one positive sample. This was mainly due to the presence of two positive samples with low toxoplasma antibody titers in panel 071. The median S/CO values for the two positive samples were 1.0 (data not shown). Although such a low ratio was never again recorded because the quality of the tests improved, another weakly positive sample (0923) in panel 092, in 2009, remained undetected by 88 participants (37.6%), with a median S/CO value of 1.2. The results from these panels show that there were problems detecting low titers of toxoplasma-specific IgM antibodies. When the results for panel 071 are not taken into consideration, the overall ratio of laboratories with EQA score for toxoplasma-specific IgM detection ≥ 80, ranged from 84.3% to 99.6%, with a mean of 95.48%.

**Table 3:** Overview of the IgM EQA Test Results.

			EQA score = 100		EQA score≥80		EQA score <80	
Year	Panel ID	No. of labs that reported results	No. of labs	Ratio (%)	No. of labs	Ratio(%)	No. of labs	Ratio(%)
2004	041	79	56	70.9	19	24.1	4	5
	042	82	64	78	14	17.1	4	4.9
2005	051	112	77	68.8	30	26.8	5	4.4
	052	115	79	68.7	33	28.7	3	2.6
2006	061	155	91	58.7	56	36.1	8	5.2
	062	152	127	83.6	22	14.5	3	1.9
2007	071	172	78	45.3	21	12.2	73	42.5
	072	164	135	82.3	23	14	6	3.7
2008	081	187	146	78.1	40	21.4	1	0.5
	082	194	186	95.9	7	3.6	1	0.5
2009	091	241	218	90.5	14	5.8	9	3.7
	092	234	145	62	88	37.6	1	0.4
2010	101	318	250	78.6	18	5.7	50	15.7
	102	318	286	89.9	4	1.3	28	8.8
2011	111	397	242	61	100	25.2	55	13.8
	112	399	367	92	4	1	28	7
2012	121	460	437	95	18	3.9	5	1.1
	122	469	442	94.2	9	1.9	18	3.9
2013	131	569	533	93.7	24	4.2	12	2.1
	132	567	536	94.5	27	4.8	4	0.7

### Analysis of the toxoplasma-specific IgG scheme

In general, the scores for IgG test results in the EQA scheme were better than those for IgM results (Table 4). The overall coincidence rates for negative samples (99.1%) were higher than those for positive samples (94.5%). The ratio of laboratories that correctly identified all five samples within a panel ranged from 61.1% (panel 112) to 99.3% (panel 121). Over the period of six years, more than 93.5% of the participating laboratories achieved a passing score ≥ 80, with a mean of 98.3%. Although the EQA program demonstrated an encouraging improvement of the years in accuracy for toxoplasma-specific IgG tests, unfortunately, detecting low levels of toxoplasma IgG antibody remains problematic for different assays as indicated by the median S/CO values of 1.1 (panel 112, data not shown). Of two weakly positive samples included in panel 112, 90 laboratories (36.9%) failed to detect one.

**Table 4:** Overview of the IgG EQA Test Results.

			EQA score = 100		EQA score≥80		EQA score < 80	
Year	Panel ID	No. of labs that reported results	No. of labs	Ratio(%)	No. of labs	Ratio(%)	No. of labs	Ratio(%)
2008	081	94	73	77.7	18	19.1	3	3.2
	082	93	89	95.7	2	2.2	2	2.1
2009	091	121	114	94.2	6	5	1	0.8
	092	124	120	96.8	2	1.6	2	1.6
2010	101	169	155	91.7	3	1.8	11	6.5
	102	176	164	93.2	10	5.7	2	1.1
2011	111	234	231	98.7	0	0	3	1.3
	112	244	149	61.1	90	36.9	5	2
2012	121	299	297	99.3	2	0.7	0	0
	122	308	292	94.8	13	4.2	3	1
2013	131	394	383	97.2	8	2	3	0.8
	132	410	404	98.5	6	1.5	0	0

### Analysis of methods used in EQA

The diagnostic assays used by participants during the EQA, over ten years in the case of IgM and over six years in the case of IgG, are summarized in Tables 5 and 6, respectively. Overall, the coincidence rates of the different methods for negative samples were better than the coincidence rates for positive samples. The results showed that the detection rates for positive samples for IgG and IgM differed significantly among the various methods (Chi-square test, p < 0.05). ELISA and CIA were the most commonly used methods. Over the total period during which the EQA scheme was conducted, ELISA was used by 75.2% (634/843) participating laboratories for IgM detection and by 70.8% (390/551) laboratories for the detection of IgG. The second most commonly used method was CIA, which was used by 15.3% (129/843) of the laboratories for IgM detection and by 20% (110/551) for IgG detection. The coincidence rates for toxoplasma-specific IgM- and IgG-positive samples detected by these two methods were greater than 86.6% and 94.1%, respectively. It is worth noting that the dot-immunogold filtration assay (DIGFA) misidentified the most positive samples, with coincidence rates for IgM- or IgG-positive samples of 59.3% and 25.0%, respectively.

**Table 5:** Methods Used by Participants in the External Quality Assessment for the Detection of IgM.

Methods	PT score	Coincidence rate for positive samples (%)	Coincidence rate for negative samples (%)	Total coincidence rate (%)	No. of participants using this method*
Enzyme linked immunosorbent assay (ELISA)	95	86.6	98.5	95.4	634
Dot-immunogold filtration assay (DIGFA)	89	59.3	96.9	89.1	20
Chemical immunofluorescent test (CIA)	98	96.3	98.9	98.2	129
Electrochemiluminescense assay (ECLA)	98	100.0	98.6	98.9	10
Enzyme immunochemistry luminescence assay (ECA)	90	72.2	96.6	90.4	19
Western blot or recombinant immunoblot assay (WB or RIBA)	92	75.9	96.5	92.2	15
Electrochemiluminescence (ECL)	100	100	100	100	16
Total					843

**Table 6:** Methods Used by Participants in the External Quality Assessment for the Detection of IgG.

Methods	PT score	Coincidence rate for positive samples %	Coincidence rate for negative samples %	Total coincidence rate %	No. of participants using this method*
Enzyme linked immunosorbent assay (ELISA)	98	94.9	99.2	98.3	390
Dot-immunogold filtration assay (DIGFA)	93	25.0	98.4	93.8	5
Chemical immunofluorescent test (CIA)	98	94.1	99.4	98.2	110
Electrochemiluminescense assay (ECLA)	96	100.0	95.4	96.5	9
Enzyme immuno chemistry luminescence assay (ECA)	95	93.0	96.1	95.4	16
Western blot or recombinant immunoblot assay (WB or RIBA)	96	94.4	96.9	96.6	8
Electrochemiluminescence (ECL)	100	100.0	100.0	100.0	13
Total					551

* No. of participants means non-repetitive participants using methods over the EQA scheme period.

### Analysis of enzyme-linked immunosorbent assay

As mentioned above, ELISA was the most widely used detection method in the EQA scheme. In 2003, at the start of the IgM scheme, 21 different ELISAs were used for the detection of IgM, of which 17 were Chinese domestic ELISA kits. Currently, 25 different ELISAs are being used of which 16 are Chinese domestic kits. In 2008, 19 ELISA kits were used by participating laboratories for the detection of IgG. By 2013, the number of ELISA kits increased to 23. The assays currently used by at least five participants in the EQA and the accumulative total coincidence rate, for the detection of toxoplasma-specific IgM and IgG, are shown in Table 7. The results of Table 7 also illustrated that for IgG antibodies testing the total coincidence rates of domestic test and international test performed across the 5 participating labs were all larger than 94.9%, and for IgM antibodies testing the total coincidence rates of domestic test and international test performed across the 5 participating labs were all larger than 94.4% except for one domestic test (89.9%, EIA, Modern biological technology co., LTD, Beijing, China). In general, the domestic tests perform well against the international tests according to the results of total coincidence rate.

**Table 7:** Assays Currently Used by Participants in the External Quality Assessment.

Asssay/manufacturer	IgM-specific antibodies		IgG antibodies	
	no. of participants using assay^a^	total coincidence rate^b^(%)	no. of participants using assay^a^	total coincidence rate ^b^ (%)
EIA,Antu biological engineering co., LTD, Zhengzhou,China	51	98.3	50	99.1
EIA,Hydratight biological pharmaceutical co., LTD, Zhuhai,China	9	95.7	9	99.0
EIA,Modern biological technology co., LTD, Beijing,China	28	89.9	4	94.9
ROCHE Diagnostics, Mannheim, Germany	10	100.0	6	99.0
EIA,EUROIMMUN, Lubeck, Germany	19	95.7	11	99.1
EIA, Trinity Biotech Plc, Bray, Ireland	32	96.7	29	98.5
LIAISON Toxo, DiaSorin S.P.A. Saluggia(VC),Italy	12	99.5	11	98.8
EIA,AiKang biological technology (hangzhou) co., LTD, Hangzhou, China	6	97.6	11	98.5
Architect,Abbott Diagnostics, Wiesbaden, Germany	10	99.6	7	98.8
AxSYM, Abbott Diagnostics, Wiesbaden, Germany	9	88.8	9	93.6
EIA, Adalti Inc, Italy	11	98.1	5	98.0
ELISA classic, Virion-serion, Würzburg,Germany	28	97.9	11	99.0
EIA,Beijing bell biological engineering co., LTD, Beijing, China	57	94.4	44	97.8
EIA, DIESSE Diagnostica Senese S.P.A. Milano,Italy	15	98.5	10	99.7
EIA,Medson inc., New Jersey, UK	41	99.1	22	97.5
Total no. of assays	15		15	

^a^ Assays used by participants in 2013 EQA scheme.

^b^ The total coincidence rate is accumulative year by year and the start usage years of different kits used by participants might be different.

**Fig 1:**
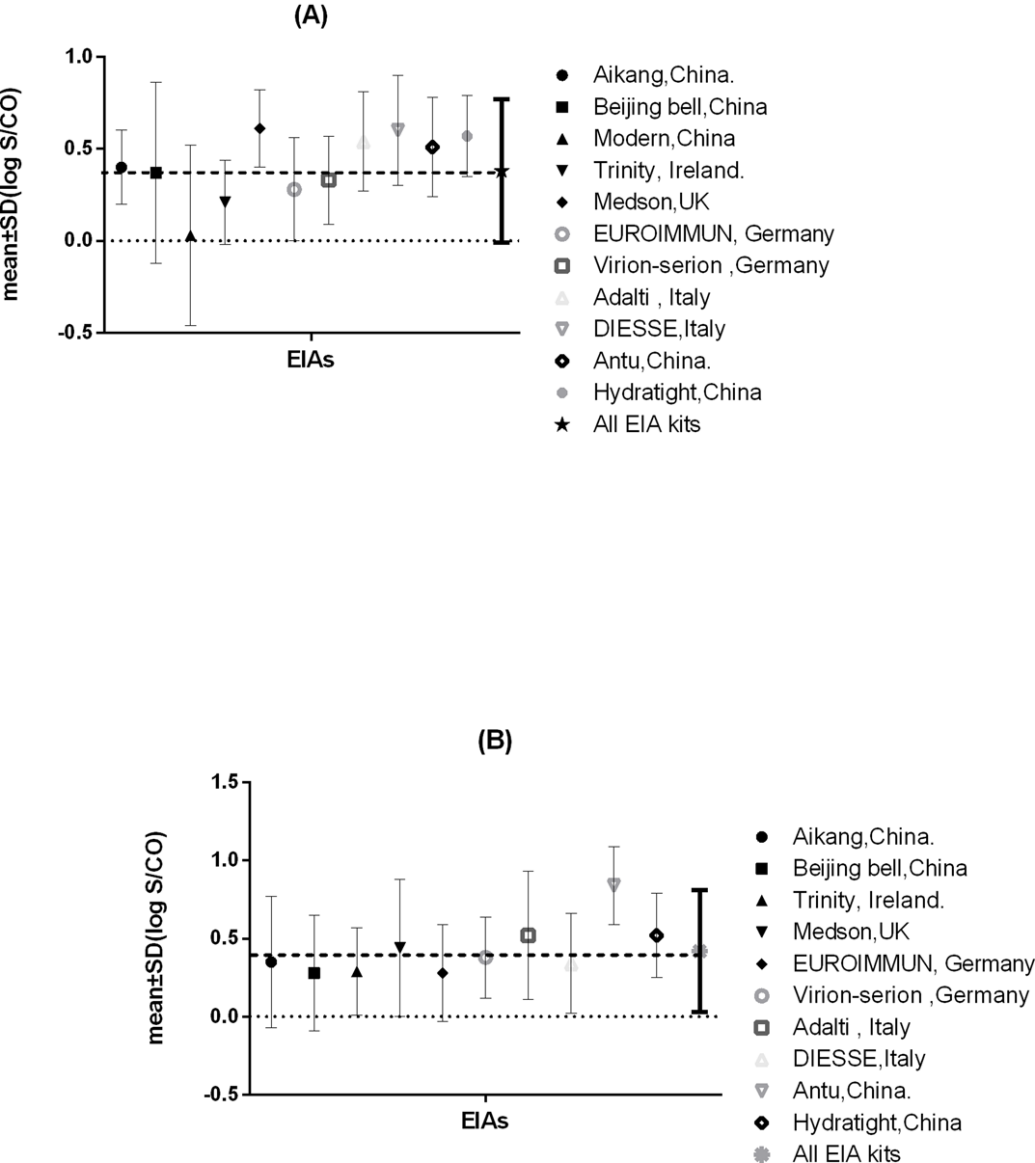
Inter-assay Variability of Assays Distributed during the EQA Scheme Period. (A) Mean (log S/CO) ± SD for each assay used by more than 5 laboratories were compared for all positive IgM specimens (B) Mean (log S/CO) ± SD for each assay used by more than 5 laboratories were compared for all positive IgG specimens. SD below the dotted line at 0.0 refers to false-negative assay results. The solid dark line in the right refered to mean and SD of log S/CO values for all assays as reference. The S/CO values for all positive samples used by each assay showed a skewed distribution, therefore, the results were transformed to the log normal distribution for analysis (P-P plot).

To determine the inter-assay variability of EIA kits from different manufacturers that are currently used in the EQA by more than five laboratories, the variability of log S/CO values was estimated by determining the standard deviation (SD) of the results of all participating laboratories for all the positive samples distributed in EQA scheme. The mean and SD of log S/CO values for each assay were determined based on the test results of all participants using EIAs, for the IgM-positive (Fig 1A) as well as the IgG-positive specimens (Fig 1B). The mean and SD of log S/CO values for all assays were also shown in Fig 1 as reference. For the IgM detection assays, results for the IgM ELISA kit (Ai Kang, Hangzhou, China) (0.4 ± 0.2 log units) and the IgM ELISA kit (Viron/Serion, Würzburg,Germany) (0.33 ± 0.24 log units) were better than those for other kits, as their mean values were closest to the overall mean (0.38 ± 0.39 log units), while their SDs were smaller than for other kits. Participants using the IgM ELISA kit (Modern, Beijing, China) reported the highest number of false-negative results (0.03 ± 0.49 log units). The largest SDs were observed for the IgM ELISA kit (Beijing Bell, Beijing, China) (0.37 ± 0.49 log units) and the IgM ELISA kit (Modern, Beijing, China). The mean log S/CO value obtained for the IgM ELISA kit (Medson inc., New Jersey, UK) was the highest (0.61 ± 0.21 log units). Fig 1B illustrates the mean and SD of log S/CO values for the IgG assays. The mean log S/CO value of the results obtained with the IgG ELISA kit (Viron/Serion, Würzburg,Germany) (0.38 ± 0.26 log units) was closest to the overall mean (0.42 ± 0.39 log units) and SDs were smaller than for other kits. The IgG ELISA kit (Antu, Zhengzhou,China) had the highest mean log S/CO value (0.84 ± 0.25 log units) among the ten EIA kits analyzed. The participants using the IgG ELISA kit (Beijing Bell, Beijing,China) (0.28 ± 0.37 log units) and the IgG ELISA kit (Ai Kang, Hangzhou, China) (0.35 ± 0.42 log unit) reported the highest number of false-negative results. The largest SDs were observed for the IgG ELISA kit (Medson, America) (0.44 ± 0.44 log units) and the Aikang assay kit.

**Fig 2:**
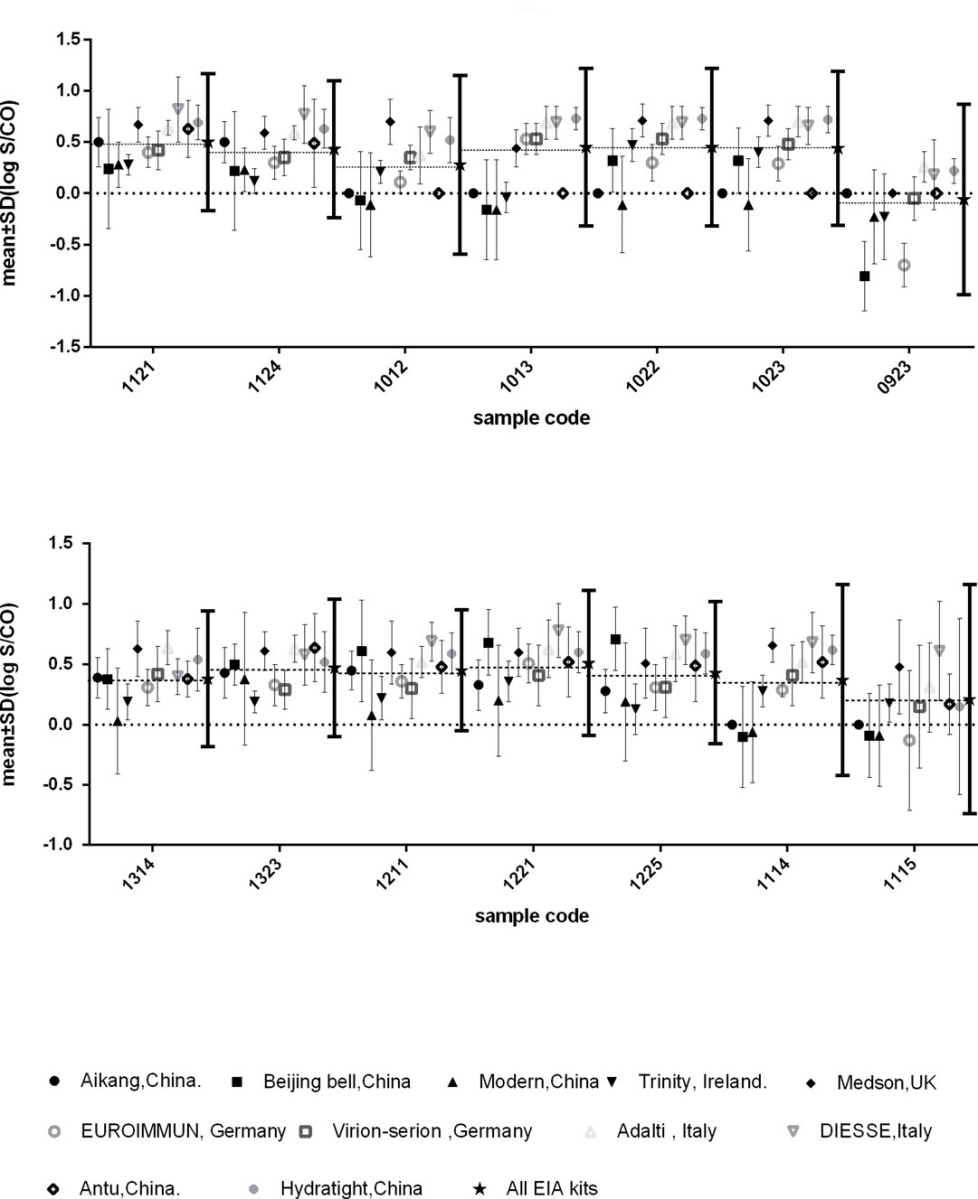
Intra-assay Variability of EIAs Commonly Used in the EQA Scheme (IgM). Mean (log S/CO) ± SD for all assays are given per sample. Absence of error bars means that the assay was used by less than 5 laboratories. SD below the dotted line at 0.0 refers to false-negative assay results. The S/CO values for all positive samples used by each assay showed a skewed distribution, therefore, the results were transformed to the log normal distribution for analysis (P-P plot).

**Fig 3:**
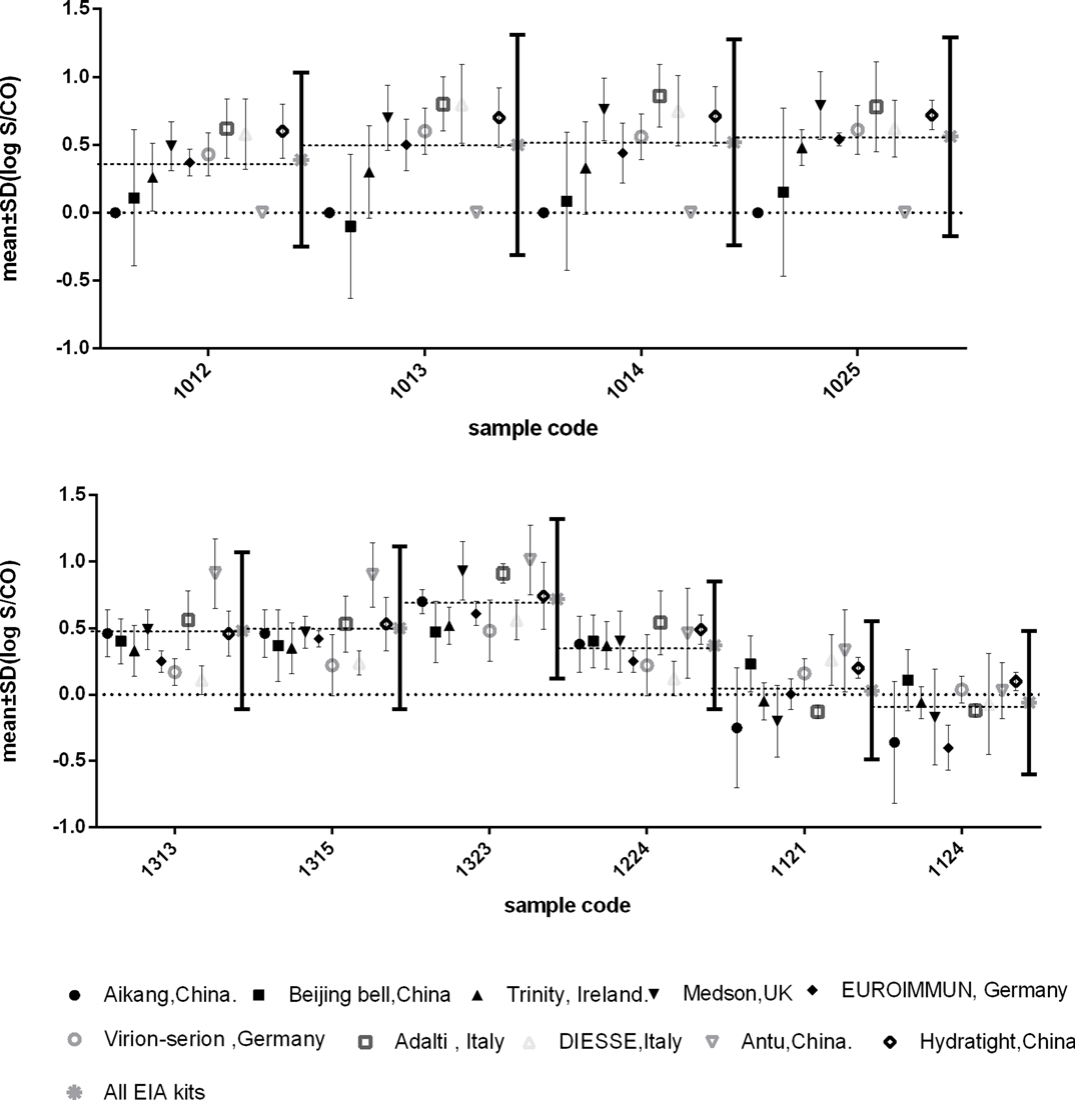
Intra-assay Variability of EIAs Commonly used in EQA Scheme (IgG). Mean (log S/CO) ± SD for all assays are given per sample. Absence of error bars means that the assay was used by less than 5 laboratories. SDs below the dotted line at 0.0 refer to false-negative assay results. The S/CO values for all positive samples used by each assay showed a skewed distribution, therefore, the results were transformed to the log normal distribution for analysis (P-P plot).

The intra-assay variability for kits currently used by more than five participants was analyzed by comparing the assay results for individual positive samples distributed in EQA scheme. Results (mean (log S/CO) ± SD) for the IgM assay kits are given in Fig 2, and for the IgG assay kits in Fig 3. The results of individual positive samples for different kits were compared with the overall average result for all EIA kits (mean (log S/CO) ± 1.96 SD). Although the accuracy of IgG and IgM detection assays had improved during recent years[2], the present results demonstrated significant variability among test results from different EIA kits used for toxoplasma-specific IgG and IgM detection. For some samples, the mean (log S/CO) for various kits deviated far from the average mean of all kits. This was observed for the IgM assay kits (Fig 2) as well as for the IgG assay kits (Fig 3). Although the highest SDs for all kits were below +1.96 SD of the average for all individual samples, the lowest SD values were observed beyond -1.96 SD for some kits. It should be noticed that false-negative results were more frequently obtained for weakly positive samples (samples 0923, 1013,1114, and 1115 for IgM, and samples 1121 and 1124 for IgG). For these weakly positive samples, Tables 1 and 2 also illustrated the coincidence rates of the participating labs using the same tests used to produce the EQA panel were not good compared with other positive samples’. The lowest coincidence rates for IgG testing was 21.4%(6/28) by Medson EiA kit for IgG sample 1121 and the lowest coincidence rates for IgM testing was 60.0%(6/10 by Trinity EiA kit for IgM sample 0923.

## Discussion

In this paper, we described the results of a long-term EQA scheme to evaluate the various immunoassays for the detection of toxoplasma-specific IgG and IgM used by the participating laboratories. Conducting such a program is the best method to detect problems with regard to assay sensitivity and accuracy, and to maintain and improve the quality of routine toxoplasmosis diagnosis in clinical laboratories.

The primary goal of EQA is to assess the participants’ diagnostic assay performance. A UK National EQA scheme for toxoplasma-specific IgG was established in 1993, followed by a scheme for toxoplasma-specific IgM in 1998[7]. These EQA schemes revealed that the performance of participating laboratories was consistently high, with a mean accuracy of 98% for IgG and of 95% for IgM. The Chinese National EQA scheme for toxoplasma-specific IgM was established in 2003, followed by a scheme for toxoplasma-specific IgG in 2008. If the strongly deviating results for panel 071 for IgM detection in the current study are not taken into account, the overall EQA score for toxoplasma-specific IgM and IgG detection in Chinese laboratories was consistently high, with a mean accuracy of 95.48% and 98%, respectively, similar to the results obtained in the UK study. Over the 10-year period during which this EQA was conducted, participation in the scheme increased, reflecting the increasing awareness of the importance of accurate diagnostic tests for an early detection of toxoplasma infection in order to appropriately treat infected individuals.

Although the overall high EQA scores in the current EQA program demonstrated an encouraging improvement in the accuracy of toxoplasma-specific IgG and IgM assays, we found a large variability in the assay results of the participating laboratories. For example, some of the participants reported false-negative results for some samples, while other participating laboratories correctly identified the same samples, using the same assay kit (Figs 2 and 3). One reason for the observed large variability could be measurement errors made by the participants during assay detection or in the selection of the cut-off value. The comparative test scores provide low-performance laboratories with the opportunity to examine their weaknesses and improve their methodologies. Participation in an interlaboratory comparison programme, such as EQA or PT(proficiency testing), is significant for clinical laboratories according to ISO 15189(Medical laboratories-Requirements for quality and competence) [8]. It allows for participating laboratories assess whether their testing results are comparable with other clinical laboratories testing results, and the participating laboratories should monitor the results of EQA and implement correctives action to improve its performance levels when their results are discrepant with expected results. Previous study domenstrated that assay interassay CVs for positive control were from 2.7% to 22.2% for six commercial kits for detection of IgM antibodies to toxoplasma gondii testing, while intra-assay precision varied from 4.9 to 13.4% for the manual assays[9]. Therefore, another reason might be the variability among kit lots from the same manufacturer. Our present study illustrated that the variability might exist in IgG and IgM testing. A clinical laboratory must institute its own verification procedures for each kit lot to ensure a continuing supply of comparable lots of reagents.

There is wide variety of assays available for the detection of toxoplasma-specific IgM and IgG. Some analyses based on commercial assay are necessary, because it is a key factor in clinical determination. Tough other techniques, such as Sabin-Feldman dye test(DT), latex agglutination test (LAT)for toxoplasma specific IgG detection, immunosorbent agglutination assay (ISAGA) for toxoplasma specific IgM detection, are mostly reserved for reference laboratories, while ELISA is mostly used in clinical laboratories for routine screening for IgG and IgM. ELISA and CIA are available as commercial kits and on automated platforms [10]. ELISA and CIA were the most commonly used methods in the laboratories that participated in this EQA scheme. Therefore, the panels with serum specimens containing toxoplasma IgG and IgM antibodies used in the EQA were tested by NCCL prior to dispatch, using various ELISA or CIA kits. Few participating laboratories used other techniques such as ECLA, ECA, ECL, DIGFA, and WB. However, the overall coincidence rates for IgM- and IgG-positive samples detected by these methods were lower than for ELISA and CIA. Especially DIGFA misidentified most positive samples. This demonstrates the different sensitivities of various methods and the importance of choosing an accurate and sensitive assay for clinical diagnosis.

Most commercial serological kits use native antigens prepared from tachyzoites grown in mice and/or tissue culture. The preparation methods for these antigens vary in different companies and the produced antigens may contain contaminants from culture media and eukaryotic host cells [10]. The major drawback is the poor standardization due to variation in antigen quality and subsequent variability in test results, which often leads to misinterpretation of the results [11]. Previous study[12] evaluated four commercial immunoassay systems and found that overall agreement rates among the four immunoassay systems were 91.7% for toxoplasma IgG and 89.8% for toxoplasma IgM. Especially the predictive value of IgM positivity strongly varies from one assay to another [13]. Therefore, we made a detailed comparison of S/CO values for different commercial ELISA kits. We found a large inter-assay variation for different ELISA kits for IgG and IgM detection (shown in Fig 1). This variation might be explained by the use of different antigens in various kits, with different antibody specificities. This complicates standardization of commercial kits. The lack of quality control and low specificity and/or sensitivity of many commercial kits form the main problems in serologic diagnosis of toxoplasmosis [3].

A detailed comparison of different commercial ELISA kits in the current study showed that some kits did not sufficiently allow for detecting weakly positive samples (Figs 2 and 3). The assay performance for panel 071 for IgM detection was rather low (42.5% failed to detect two or more positive samples and 12.2% missed one or two positive samples), and 88 participants (37.6%) failed to correctly identify sample 0923 in panel 092. Although these low scores never occurred again later in the EQA scheme, these results are noteworthy. The lower titers of toxoplasma antibodies in these samples could explain the poor assay results. The assay performance results for this sample in Fig 3 also illustrated this problem for IgG testing. In clinics, a false-negative result can lead to misinformation of the patients, which can be especially fatal for fetuses when pregnant women are infected. Therefore, the manufacturers should thus pay attention to the sensitivity problem of the tests.

Most ELISAs can detect IgM for months or years after infection, so IgM detection is not an accurate marker of recent infection [14]. The differences in IgM in acute cases and chronic cases are lower levels of the antibody in the latter and different molecular weights of the antibodies. The low molecular weight, 7s IgM is more common in persistent infection while the 19s IgM is more common in acute infection; and may help in differentiating acute from persistent infection [15]. Because of the high titer of most IgM positive samples, the sera came from acute cases. The detection principles of EIAs or CIAs for IgM are capture method or indirect method aiming at the detection of IgM, therefore, these reagents could not distinguish 7s IgM or 19s IgM and these reagents could not recognize chronic IgM or acute IgM. When confirming an acute IgM result, additional tests such as the determination of IgG avidity are currently used in clinical laboratories [2,16]. However, specimens for IgG avidity testing were not included in the present EQA scheme and should be also included in future EQA schemes in order to discriminate between recent and past infection.

In conclusion, the long-term national EQA toxoplasma serology scheme in China has been beneficial to the participating clinical laboratories; the IgG and IgM assay performance improved and the sensitivity of routine tests as well as the accuracy of assay data analysis increased during the past years, thanks to the educational approach of the EQA scheme. This shows that a continuous monitoring of laboratories by an EQA can improve national performance, underlining the importance of such EQA.

## Supporting Information

S1 Table Assays Currently Used by less than 5 Participants in the External Quality Assessment. (DOCX)
