# Supplementary material for: Results of the National External Quality Assessment for Toxoplasmosis Serological Testing in China
Source: PLoS One. 2015 Jun 12;10(6):e0130003. doi: 10.1371/journal.pone.0130003 (PMC4466578; doi:10.1371/journal.pone.0130003)
Supplement: S1 Table — (DOCX) [file pone.0130003.s001.docx]

**Supplementary material**

**Table S1 Assays Currently Used by less than 5 Participants in the External Quality Assessment.**

| Asssay/manufacturer | IgM-specific antibodies | | IgG antibodies | |
| --- | --- | --- | --- | --- |
|  | no. of participants using assay | total coincidence rate(%) | no. of participants using assay | total coincidence rate(%) |
| EIA, Shanghai Kehua Biotechnology Industrial Co., Ltd., Shanghai,China | 1 | 86.7 | 1 | 100 |
| EIA, Beijing Kewei clinical diagnostic reagent factory, Beijing ,China | 1 | 100 | 1 | 100 |
| EIA,Shandong Weifang 3V diagnostic technology company, Weifang,China | 1 | 91.1 | 1 | 100 |
| EIA,UW GBI biotech (Beijing) Ltd. , Beijing ,China | 1 | 100 | 1 | 98.0 |
| EIA,Biomerieux,SA, Marcy,France | 1 | 100 | 1 | 100 |
| EIA,Zhuhai Lizhu reagent Limited by Share Ltd , Zhuhai ,China | 1 | 98.0 | 1 | 100 |
| EIA,Zeus Scientific,Inc, Branchburg ,America | 4 | 91.8 | 4 | 99.5 |
| EIA,CanAg Diagnostics AB, Goteborg City, Sweden | 4 | 94.1 | 0 | - |
| EIA,CITIC Shanghai Ast diagnostic reagents Ltd. , Shanghai,China | 1 | 97.0 | 0 | - |
| EIA,Beijing source of Biomedical Engineering Ltd. , Beijing ,China | 4 | 96.5 | 4 | 97.0 |
| EIA,Zhengzhou Bosai biotech Limited by Share Ltd, Zhengzhou, China | 1 | 100 | 1 | 96.7 |
| EIA,Weifang Hong Wah biotechnology limited company, Weifang,China | 4 | 91.1 | 4 | 94.3 |
| EIA,Zhuhai Huaao biotechnology Ltd. , Zhuhai ,China | 1 | 100 | 1 | 100 |
| EIA, Born in becom biological Polytron Technologies Inc, Beijing ,China | 1 | 100 | 1 | 100 |
| Total no. of assays | 14 |  | 12 |  |
